# Assessment of renal glomerulosclerosis and thickness of the carotid intima-media complex as a means of age estimation in Western European bodies

**DOI:** 10.1007/s00414-021-02705-w

**Published:** 2021-11-13

**Authors:** Carl Daniel Lehmann-Leo, Frank Ramsthaler, Christoph G. Birngruber, Marcel A. Verhoff

**Affiliations:** 1 Institute of Legal Medicine, University Hospital of Frankfurt, Goethe University, Kennedyallee 104, 60596 Frankfurt/Main, Germany; 2 Department of Anesthesiology, Operative Intensive Care Medicine and Pain Therapy, University Hospital Gießen and Marburg, Gießen, Germany; 3 Institute of Legal Medicine, University of Saarland, HomburgSaar, Germany

**Keywords:** Forensic pathology, Age estimation, Biological profile, Sclerotic glomeruli, Glomerulosclerosis, Intimal-medial thickness

## Abstract

**Introduction:**

The estimation of age-at-death of unidentified cadavers is a central aspect of the identification process. With increasing age, the incidence of glomerulosclerosis and the thickness of the carotid wall have been observed to also increase. This correlation has been demonstrated in various international histological studies. The aim of our study was to assess whether these correlations also apply to a Western European population.

**Methodology:**

In this retrospective observational study, kidney and common carotid artery samples from 216 cases autopsied at the Institute of Legal Medicine at the Justus-Liebig University in Giessen, Germany, were examined. Only cases with available tissue samples from both body sides were included. Exclusion criteria were poor sample quality and an age younger than 21 years. After histological processing, the tissue samples were assessed and digitally evaluated. Regression and classification analyses were used to investigate the correlation between age-at-death and intima-media thickness and age-at-death and the incidence of renal glomerular sclerosis.

**Results:**

Of the 216 autopsy cases, 183 were included for evaluation. Analysis of the carotid artery segments showed a strong correlation (Pearson correlation coefficient *r* = 0.887) between the intima-media-complex thickness and chronological age. Classification of the glomerulosclerotic incidence showed a correlation of 37.7–43.1% with the predicted age group.

**Discussion:**

Both the intima-media thickness and the proportion of sclerotic glomeruli can be used to estimate age in Western European cadavers. On the basis of these results, both methods are suited to supplement other already established methods for age-at-death estimation in the identification of an unknown cadaver.

**Supplementary Information:**

The online version contains supplementary material available at 10.1007/s00414-021-02705-w.

## Introduction

As part of the forensic procedure to identify unknown decedents or victims of mass disasters, a “biological profile” is compiled. Age-at-death is one of the key characteristics of this biological profile, which also includes data on sex, ancestry, stature, and the documentation of existing diseases [1]. With these postmortem data, potential matches can then be identified from missing persons lists.

During adolescence, up to an age of 21 years, age estimations are mainly of interest in legal contexts pertaining to criminal and asylum law [2]. Up to this age, methods that are useful for the estimation of age, in addition to anamnesis, are x-rays of the left hand (useful until about the 18th year of life) [3], panoramic dental radiographs to assess tooth mineralization status [4], and, in cases with completed skeletal growth of the hand-wrist, additional radiographic assessment of the medial epiphyseal clavicle by computed tomography [5–7]. These methods, based on changes of the growing skeleton, allow estimation of age with the requisite degree of medicolegal certainty needed to answer whether or not a legally relevant chronological age has been attained. Additionally, these methods can be helpful in estimating age-at-death in the identification of an unknown cadaver. Beyond the end of puberty, with complete ossification of the skeleton, which occurs approximately around the 21st to the 23rd year of life, and the end of growth-dependent changes of the body, these methods are no longer of use. From this point on, in contrast to age estimation in juveniles, age-estimation methods based on degenerative changes and time-dependent effects on the body increasingly gain prominence for adults [8]. Moreover, the emphasis of age estimation in adults shifts from the determination of attained legally relevant age thresholds towards the identification of unknown decedents. Once skeletal growth has ceased, other methods for age estimation are therefore employed, such as assessment of a combination of several morphometric bone characteristics [9, 10], e.g., pubic symphysis [11, 12] and sternal rib ends [13], or methods based on various radiological and macroscopic dental characteristics [14–16].

More accurate age estimates in adults than those provided by classic anthropological methods can be achieved by measuring the racemization of aspartic acid in dentin [17, 18] or by determining incremental lines of dental cementum [19–21]. In addition, methods that allow age estimation on the basis of histological changes in bone structure have been published. Another more recent method from the field of epigenetics looks at age assessment on the basis of DNA-methylation markers [22–27].

Because these different methods have different estimation errors (histological bone features ± 5–10 years (SE, standard error) [9, 10], aspartic acid racemization in dentin ± 3 years (error) [18], telomere shortening ± 9.8 years (SEE, standard error of estimate) [28], DNA-methylation ± 3.75 years (MAD, mean absolute deviation) [26]), the accuracy of the estimated age interval can be increased by combining methods, especially by revealing discrepancies [8, 29, 30]. Moreover, it has been shown that the use of different methods, or combinations of methods, for individual age intervals delivers the best results [8, 31]. All of these methods, unfortunately, have limitations that can preclude their use on cadavers. If, as is sometimes the case in medicolegal case work, only highly decayed cadavers, isolated body parts, or tissue structures are available, it is of immense significance to be able to resort to a broad spectrum of established age-estimation methods. In this context, the quantification of age-related physiological changes in histological organ samples, such as the incidence of sclerotic glomeruli in kidneys, or increasing thickness of the intima media of the common carotid artery, appears to offer further options.

In the kidney, specific age-related changes in the glomeruli become apparent in the course of aging and can be histologically evaluated [32–35]. Due to age- and disease-related processes, there is increasing nephron rarefaction and, consecutively, loss of nephron structures. Hyaline deposits in the glomeruli become apparent and can be quantified. Because the proportion of glomeruli with hyaline deposits (sclerotic glomeruli) is known to increase with age, they are, thus, of interest as a means of forensic age estimation [36–41]. Several international studies have already demonstrated this correlation between the incidence of glomerular sclerosis and biological age [36–38, 42]. However, only one of these studies was conducted on a European population [37]. Since population-specific differences in the incidence of glomerulosclerosis are conceivable, the available data, therefore, currently precludes application of this method to a German population [36–38, 42]. Among other possible factors, in their study, Fukuda et al. discuss the effect of different lifestyles and eating habits as possible causes for such population-specific differences [42].

The results of epidemiological studies investigating atherosclerosis in cranial blood vessels showed that there was a sex-independent, age-related increase in the thickness of the carotid artery wall of 0.01 mm/year in vessel segments that were not affected by atherosclerosis [43]. In these studies, the intima-media thickness (IMT) of the common carotid artery could be determined in histological specimens as well as by sonography [43–45]. Determination of the intima-media thickness of the common carotid artery could thus also be of interest in forensic age estimations.

The purpose of our study was to analyze a selected collective of autopsy cases from Germany, in regard to age-related glomerulosclerosis and intima-media thickness, with the aim of assessing the usefulness of these two parameters, as a means of estimating age in a Western European population.

## Material and methods

**Fig. 1:**
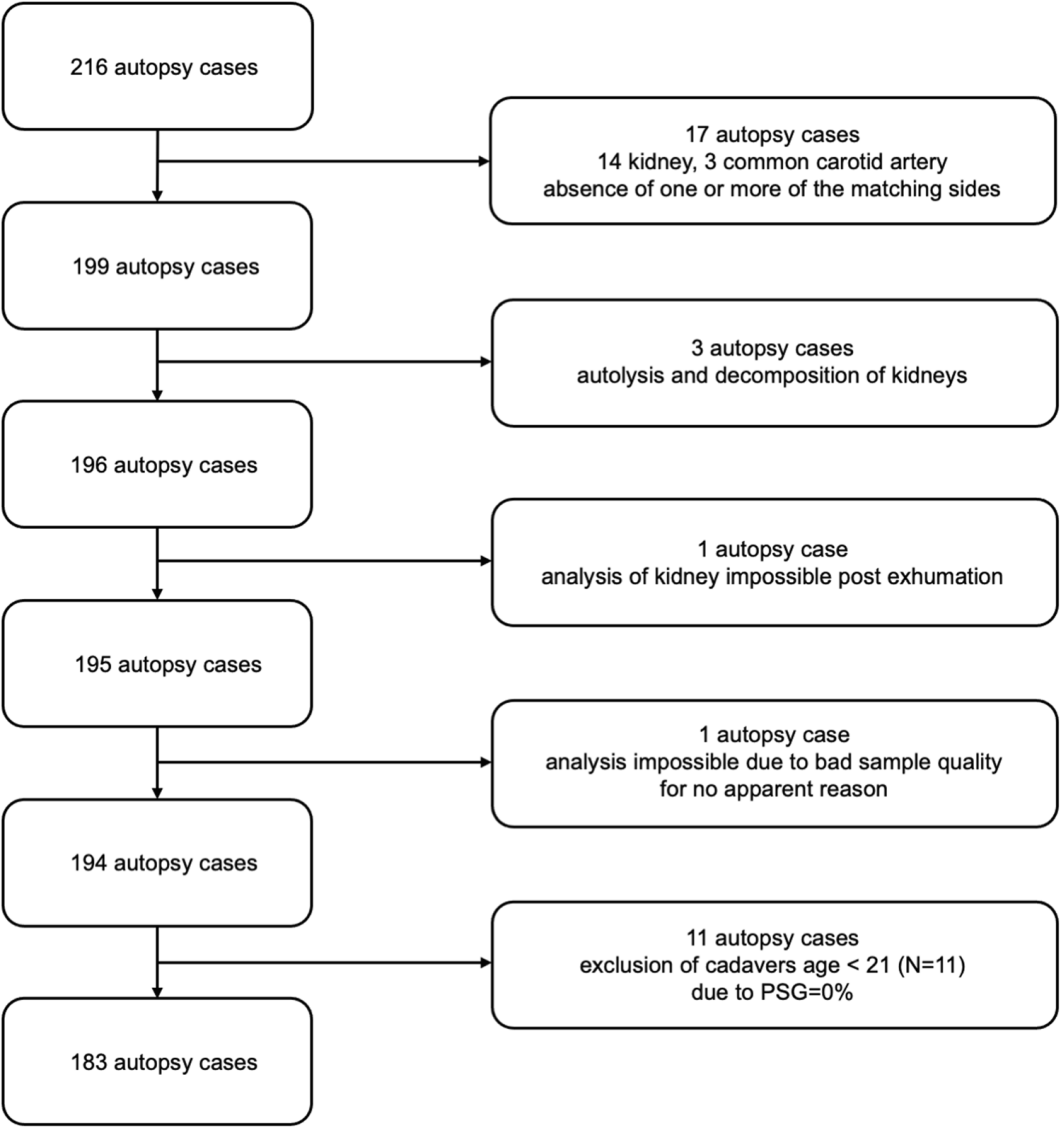
Case selection. All cases with decedents aged between 21 and 89 years for whom histological kidney and carotid samples from both body sides were available for evaluation were included

This was an observational, retrospective study conducted on cadaveric histological kidney and common carotid artery samples from 216 decedents autopsied at the Institute of Legal Medicine at the Justus-Liebig University in Giessen, Germany, from 2010 through 2011. The ethics committee of the Goethe University in Frankfurt am Main, which was consulted whether approval for the study was necessary, ruled that none was required. There was no case selection on the basis of the decedents’ age, sex, manner of death, or clinical history. The inclusion criterion for the case selection was that tissue samples from both kidneys and both common carotid arteries of the decedents were available. Tissue samples that could not be evaluated due to autolysis, putrefaction, and exhumation were excluded. Samples from decedents younger than 21 years of age were also excluded, based on the fact that the incidence of sclerosis in this age group is expected to be too low for analysis (Fig. 1).

### Sample preparation

Cadaveric tissue samples from both kidneys and both the left and right common carotid arteries were collected during forensic dissection. The tissue samples were fixed in 4% neutral buffered formaldehyde before being embedded in paraffin. The embedded samples were then sectioned and slices of 5–8 µm thickness were mounted on glass slides. Next, the paraffin was removed by passing the samples through decreasing concentrations of alcohol. The samples were then standardly stained with a hematoxylin–eosin stain, before being imaged with a light microscope (40 × magnification, light microscope BX51(TF), Olympus, Tokyo, Japan) and being digitized with a digital camera (camera DP72, Olympus, Tokyo, Japan).

### Quantification of sclerotic glomeruli

**Fig. 2:**
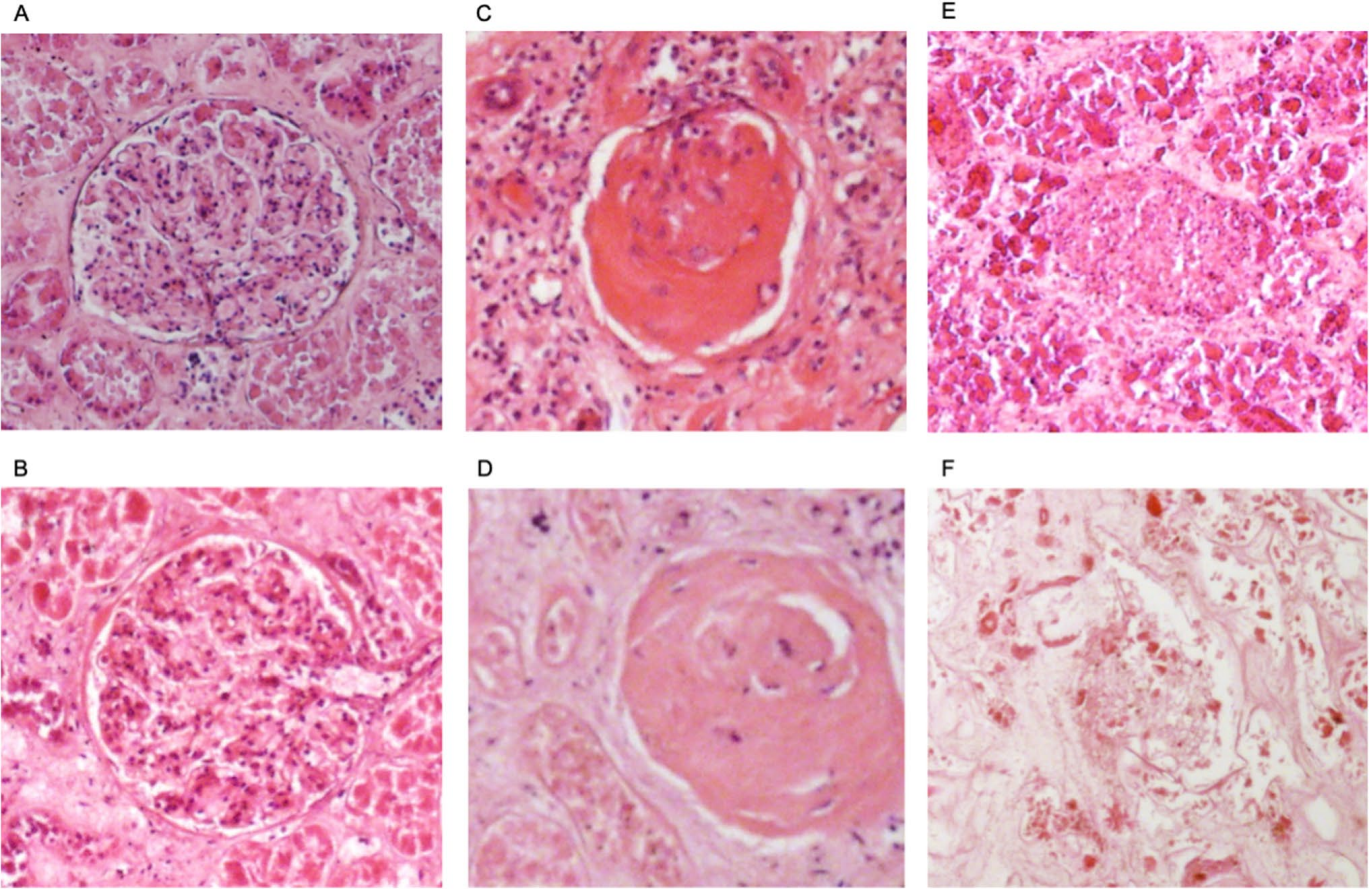
Non-sclerotic renal glomerulus (**A** + **B**); sclerotic glomerulus (**C** + **D**); renal glomerulus after putrefaction and autolysis (**E**); kidney after exhumation, PMI 492 days (**F**)

To determine the proportion of sclerotic glomeruli, a tissue sample, measuring approx. 2.5 cm × 1.5 cm, was cut from a macroscopically inconspicuous horizontal section from the middle third of the renal cortex. This procedure was done for both kidneys. Per left and right kidney, ten non-overlapping, adjacent sections from the histologically prepared samples, measuring 2.23 mm × 1.66 mm, were photographed. Next, the total number of glomeruli and the number of hyalinized (sclerotic) glomeruli were counted in the images. Glomeruli on the edge of an image were included if at least 50% of the glomerulus was still clearly visible. A sclerotic glomerulus was defined as one that was completely sclerotized or completely filled with hyalinized material. Glomeruli that were only slightly or moderately hyalinized were not counted as sclerotic for better comparison with existing studies on the topic [42]. Figure 2, for comparison, shows images of normal samples, hyalinized samples, and samples that could not be evaluated.

The samples were counted in a blinded evaluation, i.e., the examiner did not know the decedent’s age, sex, medical history, or manner of death. The numbers of sclerotic and non-sclerotic glomeruli were subsequently added together, and the quotient was determined (sclerotic/non-sclerotic glomeruli).

### Quantification of the intimal-medial thickness in carotid artery segments

To determine the IMT of the carotid arteries, a 1-cm-long vessel segment was removed immediately proximal to the carotid bifurcation, both for the left and the right carotid artery (online supplementary material). After fixation of the arterial segments, between 1 and 11 digital images were captured per side (size 2.23 mm × 1.66 mm).

**Fig. 3:**
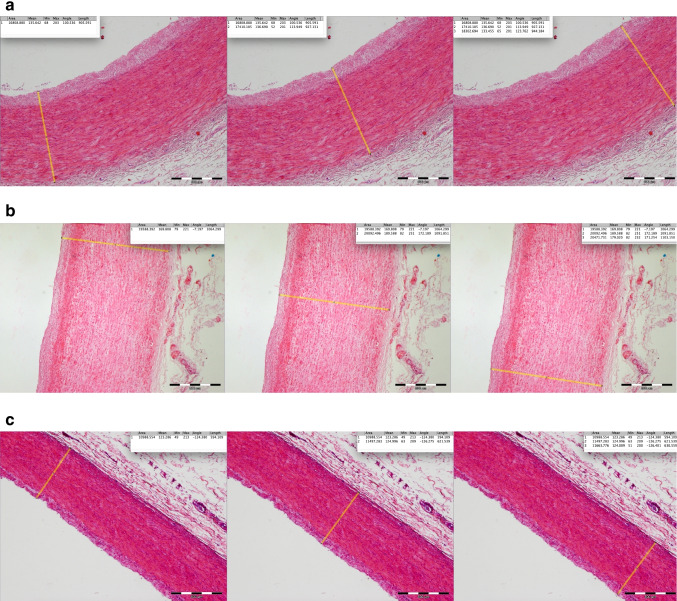
**a**–**c** Measurement of the IMT of the common carotid artery Three measurements per sample were taken and the mean value was then calculated

Each of the digital images was then subdivided into 3 equal sections, and the IMT in each section was manually measured (Fig. 3) (Fiji ImageJ Version 2.0.0-rc-69/1.52i, GitHub, San Francisco, USA). The IMT was defined as the distance between the vessel lumen (border of vessel lumen and tunica intima) and the edge of the tunica media (border of tunica media and tunica externa/tunica adventitia). The mean value for the measurements from the subsections was calculated for further evaluation.

### Statistical analysis

Statistical analysis was performed with MedCalc Software Ltd Belgien, MedCalc Version 19.0.3 (7. Mai 2019). The descriptive analysis encompassed the mean value, standard deviation, and variance.

For the statistical analysis of the hyalinized glomeruli, the histologically prepared samples were sorted by sex and body side and then grouped into 4 classes of sclerosis incidence (Class 0: No sclerosis; Class 1: > 0 to 5%; Class 2: > 5 to 10%; Class 3: > 10% sclerosis). These classes were then assigned to age groups of 21–35 years, 36–50 years, 51–65 years, and > 65 years. Subsequently, this assignment to a (predicted) age group on the basis of the above criteria was controlled by comparison with the true age-at-death of the decedent from whom the histological sample had been taken. Four sub-classes, divided by sex (male/female) and body side (left/right), were looked at.

To statistically analyze the correlation between age and IMT, the Pearson correlation coefficient and the standard error of estimation (SEE) were calculated. Furthermore, three regression models (linear, potential, and exponential regression) were investigated to describe the correlation, and the highest coefficient of determination (*R*^2^) was identified. The purpose of this statistical procedure was to identify the lowest variance in a regression model used to describe the statistical correlation between IMT and biological age.

For age estimation in the examined cases, canonical classification (discriminant function) analysis was used together with the evaluated characteristics for predicting the age group an individual belonged to.

## Results

### Basic characteristics

After application of the inclusion and exclusion criteria, cadaveric samples from 183 of 216 autopsied cases were analyzed (Table 1). In one case, the reason for the poor quality of the prepared kidney sample could not be elucidated from the documentation. The exclusion of autopsy cases due to poor quality of the processed histological samples was, in all cases, related only to kidney tissues. This exclusion criterion had no bearing on the assessment of the IMT of the carotid arteries, and the carotid tissue preparations could be evaluated for all of the 216 decedents. However, in some of the cases, carotid samples were only available from one side of the body. Retrospective assessment of our histological samples after reviewing the dissection records in regard to kidney-altering diseases and arterial hypertension did not allow the conclusion that these diseases might have affected the results of our evaluations.

**Table 1:** Demographics and characteristics of the included forensic autopsy cases

Number of cases	Total	183
	For both sides	366
Cases per age group (years)	21–35	36
	36–50	42
	51–65	47
	> 65	58
Sex	Male	130 (71%)
	Female	53 (29%)
Postmortem interval (days)	Minimum	0
	Maximum	31

The recorded postmortem interval (PMI, time since death in days) had been less than 5 days in 71.6% of the autopsy cases and above 10 days in seven of the cases (3.8%).

Table 2 depicts the descriptive statistical evaluation for the sclerotic glomeruli and the carotid IMT, without additional preselection or filters.

**Table 2:** Descriptive statistics for sclerotic glomeruli and the IMT, without filters

	**Number (*N*)**	**Minimum**	**Maximum**	**Mean value**	**Standard deviation**	**Variance**
Age	366	21	89	54.45	18.308	335.185
Number glomeruli	366	56	204	118.01	22.273	496.101
Percent glomeruli	366	0.0	96.1	3.73	6.565	43.096
Number of measurements IMT	366	3	33	16.58	5.154	26.562
IMT minimum	366	260	1090	714.94	144.722	20,944.435
IMT maximum	366	539	1631	996.68	189.142	35,774.760
IMT mean values	366	468	1241	843.51	148.585	22,077.440

The table presents the descriptive statistics for sclerotic glomeruli and IMT of the carotid artery after application of inclusion and exclusion criteria

### Correlation of sclerotic glomeruli and biological age

The histological samples from the left kidney of female cadavers were correctly classified in 37.7% of the cases [*n* = 20/53]; in the case of the male cadavers, the samples for the left kidney were correctly classified in 43.1% of the cases [*n* = 56/130]. For histological samples from the right kidney, the classification was correct for 37.7% of the female cadavers [*n* = 20/53], and correct in 40.8% of the cases [*n* = 53/130] for the male cadavers.

The classification by body side (left/right) and sex is presented in the online supplementary material.

### Correlation of the intimal-medial thickness of the carotid artery and biological age

**Fig. 4:**
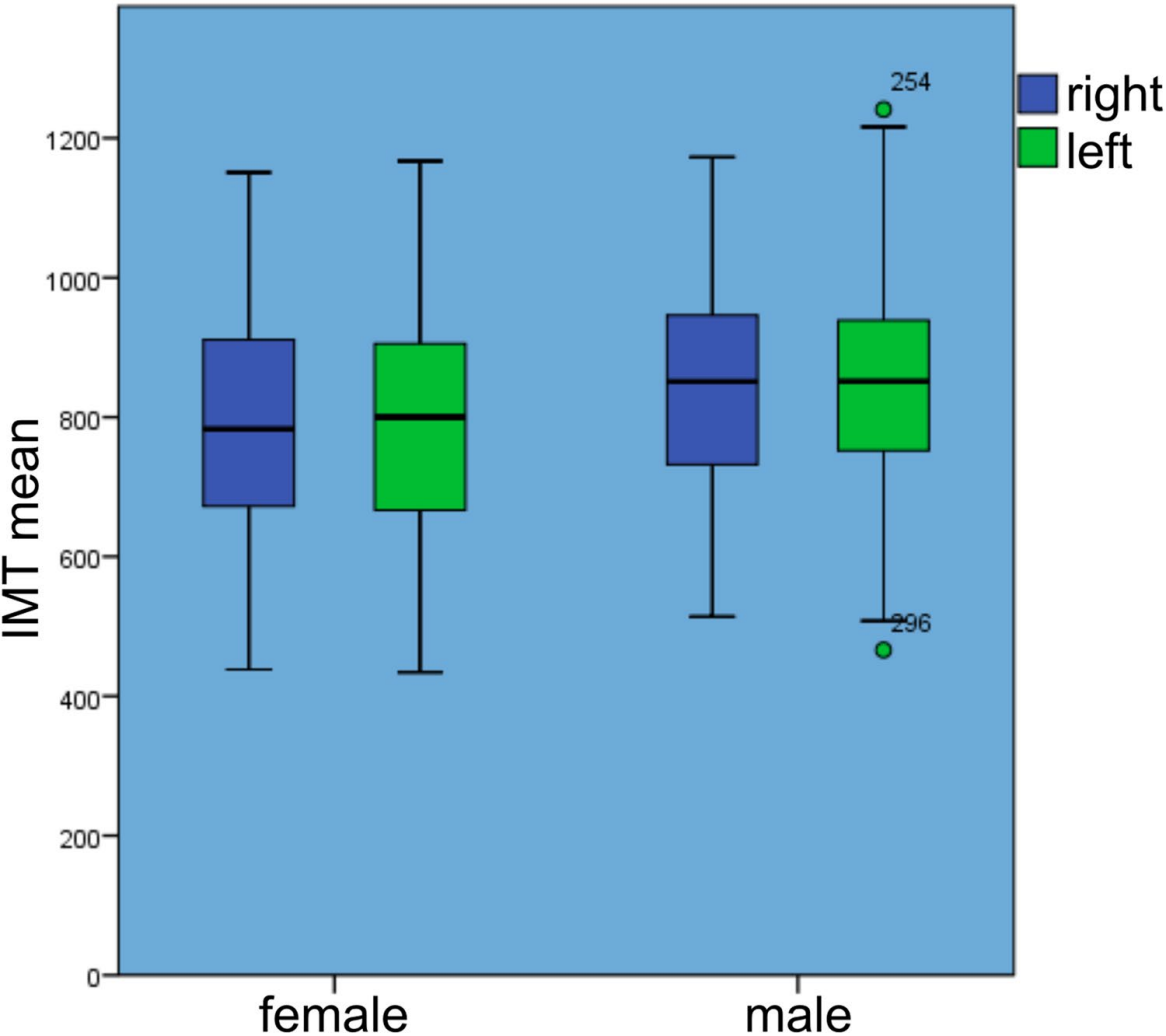
Distribution of IMD mean value according to sex

No significant difference in the IMT of the common carotid arteries could be found between the left and the right carotid. Similarly, no significant difference in IMT could be found between men and women (Fig. 4).

At a level of *p* = 0.01 (one sided) and *r* = 0.887, the Pearson correlation coefficient showed highly significant correlation between biological age of the individual and IMT.

Without application of selection criteria (i.e., all PMI, all age groups, left and right body sides together), there was a high standard estimation error (SEE: 13.9–14.0) in the regression analysis.

**Fig. 5:**
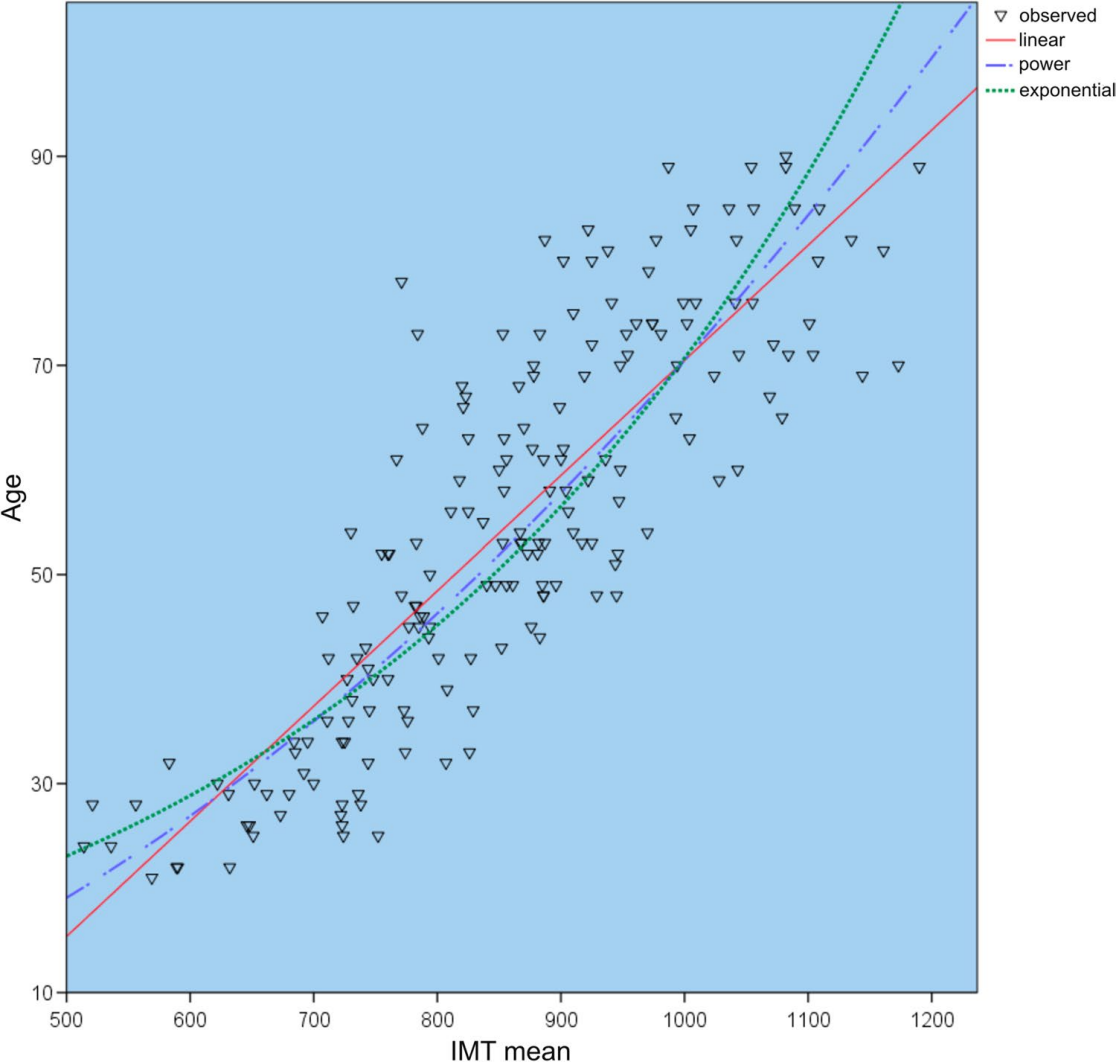
Regressions curves for the mean values for IMT and age for the right carotid artery and a PMI of less than 5 days

Better results could be achieved when the postmortem interval was restricted to less than 5 days, and the left and right body sides, and both sexes were looked at separately. For each sub-group (in total 6), three regression models (L: Linear, P: Potential, E: Exponential) were looked at and compared. Table 3 shows the results of the regressions for the various models and sub-groups. Figure 5 shows the sub-group composed of the right carotid artery and PMI of less than 5 days. The results for the six sub-groups that were analyzed did not vary significantly from each other.

**Table 3:** Regression analyses for the IMT of the common carotid artery

Selection	Number (*N*)		Regression formula	*R*	*R*^2^	*p*	SEE
Right side, PMI < 5 days	131	lin	*y* = 0.110 *x* + (− 39.725)	0.844	0.71	< 0.001	8.07
		pot	*y* = 0.0001* × 1.885	0.86	0.739	< 0.001	8.12
		exp	*y* = 7.529 * exp (0.0022*x)	0.848	0.719	< 0.001	8.55
Right side, male PMI < 5 days	99	lin	*y* = 0.106 *x* + (− 37.45)	0.83	0.69	< 0.001	8.26
		pot	*y* = 0.0001* × 1.868	0.85	0.73	< 0.001	8.36
		exp	*y* = 7.413 * exp (0.0022*x)	0.84	0.71	< 0.001	8.9
Right side, female, PMI < 5 days	32	lin	*y* = 0.122 *x* + (− 46.95)	0.887	0.78	< 0.001	6.3
		pot	*y* = 0.0001* × 1.932	0.886	0.78	< 0.001	6.35
		exp	*y* = 7,74 * exp (0.0022*x)	0.877	0.71	< 0.001	6.74
Left side, PMI < 5 days	131	lin	*y* = 0.102 *x* + (− 32.20)	0.80	0.64	< 0.001	8.5
		pot	*y* = 0.0039* × 1.749	0.81	0.66	< 0.001	8.7
		exp	*y* = 8.75* exp (0.0020*x)	0.80	0.64	< 0.001	9.1
Left side, male, PMI < 5 days	99	lin	*y* = 0.104 *x* + (− 35.32)	0.825	0.677	< 0.001	8.09
		pot	*y* = 0.0002* × 1.835	0.84	0.71	< 0.001	8.31
		exp	*y* = 7.14* exp (0.0021*x)	0.83	0.68	< 0.001	8.3
Left side, female, PMI < 5 days	32	lin	*y* = 0.099 *x* + (− 25.69)	0.78	0.59	< 0.001	8.24
		pot	*y* = 0.0012* × 1.530	0.76	0.56	< 0.001	8.4
		exp	*y* = 11.39* exp (0.0018*x)	0.75	0.56	< 0.001	8.75

The table depicts the different regression models and selections. *Abbreviations*: *R*, correlation coefficient; *R*^*2*^, coefficient of determination; *p*, significance value; *SEE*, standard error of estimation

## Discussion

The aim of this study was to investigate the correlation between renal glomerulosclerosis and age-at-death, as well as between common carotid artery intima-media thickness (IMT) and age-at-death, for a cadaver population from Germany. Forensic age estimations, which are performed in the context of compiling a “biological profile,” investigate biological age [46]. The ultimate aim of these age estimations is to be able to narrow down, or estimate, a decedent’s chronological age. Biological age itself only describes the physiological condition of an individual, which is influenced not only by genetics and environmental factors, but in the course of a life, increasingly also by lifestyle, e.g., diet, use of stimulants/alcohol, or exercise [47]. As these factors increasingly affect biological age with increasing chronological age, the discrepancy between biological and chronological age may widen, an effect that has been termed the “trajectory effect” by Nawrocki [48]. The aim in estimating the age of an unidentified cadaver is thus to use various methods to achieve the best possible agreement between biological and chronological age.

The hyalinization of renal glomeruli over the course of a lifetime is an example of such a natural, physiological process, as the incidence of sclerotic glomeruli increases with increasing age, throughout life [36–42]. While for age groups younger than 21 years old, no sclerotic glomeruli (PSG = 0%) are observed, and up to the age of 30 years, on average, only 1% of glomeruli are obsolescent. Thereafter, the hyalinization slowly increases but rarely exceeds 7% below the age of 50 years. Between the age of 50 and 66 years, PSG is usually less than 12%, whereas after the age of 66 years, the rate of sclerosis varies greatly. This seems a little different to the results of Fukuda et al. [42] in which the rate of sclerosis in the Japanese population was less than 0.6% in age groups younger than 33 years old, and a sclerosis rate of 6% with high probability indicated an age of older than 55 years. For the USA, Kaplan et al.[36] demonstrated that up to age 40 to 45 years it would be expected to have PSG values no greater than 10%; however, after the age 45 to 50 years, the observed values exceeded 10% with a broad scatter. Based on the observed differences in the individual collectives, population-specific differences appear possible due to the reasons listed above.

In our study, in which we subdivided the age span of the autopsied decedents, which had ranged from 21 to 89 years, into four groups, or age intervals, the kidney samples could be assigned to their predicted age group with an accuracy of 37.7–43.1%. Because of the high variability in estimation errors for the currently established methods, the ability by itself to narrow down a decedent’s age to a certain age group and in combination with different methods can be decisive for the positive identification of an unknown body.

The IMT of the common carotid artery is used as a biomarker for the early detection of systemic atherosclerosis. Apart from cardiovascular risk factors, chronological age is an important independent factor affecting IMT [45]. The positive correlation between age and IMT has previously been demonstrated in several epidemiological studies [43, 45, 49], and increasing IMT may represent a normal aging process of vessels [45]. Sonographic and histological measurements of the IMT have been shown to be comparable and to not differ significantly [44, 50]. These findings suggest that determination of the IMT of the carotid artery could be a means to estimate the age-at-death of an unknown cadaver. In the autopsied cadaver collective we investigated, we could also demonstrate a strong correlation between an increase in IMT of the common carotid artery and age by using different regression analyses (Pearson correlation coefficient *r* = 0.887). Despite the fact that no significant group differences were found in the IMT analysis, selection of the samples proved useful (male/female, left/right), as this increased the estimation accuracy. Although there was no statistically significant difference between the selected groups, the standard deviation of the estimation error (SEE. 6.3–9.1) could be reduced and, consequently, a better fit of the regression curves could be achieved.

In contrast to the kidney samples, histological evaluation of the carotid artery samples was possible in each of the examined autopsy cases. In the case of the five kidney samples that could not be evaluated, the poor sample quality had been mainly due to extreme external factors (warm surroundings) that had led to autolysis and putrefaction, or a very long PMI (exhumation). This suggests that the tissue from the carotid arteries was more robust towards autolysis and putrefaction than the kidney tissue. Although most of the 183 forensic autopsies included in our study had a PMI of less than 5 days (71.6%), evaluation of all tissue samples was still possible even for PMIs of longer than 10 days, provided they had not been exposed to extreme external factors. The carotid artery samples could be evaluated in every case, in as far as they were available.

The strength of the age-estimation methods we investigated here lies in the simplicity of their application, since even for incomplete cadavers, or those in a compromised condition, it is usually possible to at least obtain tissue samples from both sides of the body and to determine the sex of the decedent. As a limitation in our study, it must be noted that our methods were not tested on badly decomposed cadavers. However, it is generally agreed on that connective tissues and structures embedded in those tend to stay stable for a longer time during the process of decomposition. Therefore, we assume that they will be available for examination for a longer time frame; nevertheless, further systematic postmortem studies are required to prove this. To improve the accuracy of age estimations, the sex and ancestry of the decedent should be taken into consideration and, whenever possible, population-specific standards for age estimation should be used [51]. We could corroborate the importance of these aspects by achieving a reduction in the standard error of estimation through selection of our samples.

The information on past medical history obtained from medical examiner records and in general in the case of the identification of an unknown body is usually poorer than in hospital cases. We did not compare our limited available data with those of hospital cases. In their study, Kaplan et al. [36] demonstrated that medicolegal cases are parallel to hospital cases. Fukuda et al. [42] also stated that the correlation found in their study and in previous reports suggests that the effects of renal diseases and hypertension in medical examiner cases are negligible.

In our study, there was no case with a documented history of renal disease, diabetes, or hypertension. During our examination, only one specimen showed visible pathological changes which could be attributed to a disease.

In summary, two different methods for age-at-death estimations for Western European cadavers were investigated in this study: the IMT of the carotid artery and the incidence of glomerulosclerosis. The IMT of the carotid artery reliably demonstrated a strong correlation with biological age. A part of the histological kidney samples could also be assigned to the correct age range by classifying the incidence of sclerotic glomeruli to four age groups. Thus, both methods, on their own and in combination with each other, appear to be useful supplements to other established methods (e.g., osteological methods) for the estimation of age-at-death in the compilation of a “biological profile” for an unknown body.

## Supplementary Information

Below is the link to the electronic supplementary material.

Supplementary file1 (PDF 107 KB)

Supplementary file2 (PDF 90 KB)
