# Supplementary figures and images for: Assessment of renal glomerulosclerosis and thickness of the carotid intima-media complex as a means of age estimation in Western European bodies
Source: Int J Legal Med. 2021 Nov 13;136(3):753–63. doi: 10.1007/s00414-021-02705-w (PMC9005432; doi:10.1007/s00414-021-02705-w)

**ESM 1** Sketch of the harvest site for the evaluated common carotid artery segment

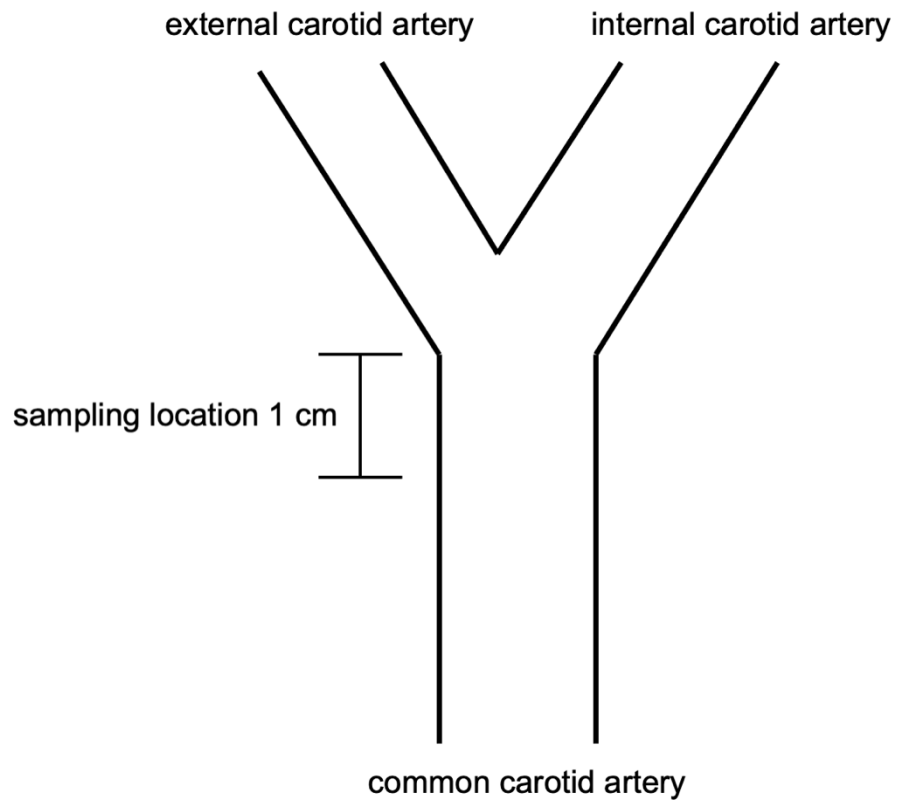

Supplement: Supplementary file 1 — Supplementary file1 (PDF 107 KB) [file 414_2021_2705_MOESM1_ESM.pdf]
