# Supplementary material for: Assessment of renal glomerulosclerosis and thickness of the carotid intima-media complex as a means of age estimation in Western European bodies
Source: Int J Legal Med. 2021 Nov 13;136(3):753–63. doi: 10.1007/s00414-021-02705-w (PMC9005432; doi:10.1007/s00414-021-02705-w)
Supplement: Supplementary file 2 — Supplementary file2 (PDF 90 KB) [file 414_2021_2705_MOESM2_ESM.pdf]

ESM 2 Classification of renal glomerulosclerosis The tables show the classification results for the kidney samples sorted by sex and body side

Selection: Left side, female		Predicted group membership (in percent of sclerotic glomeruli)				
		No sclerosis	>0% - 5%	>5% - 10%	> 10%	Total
True group membership (Age in years)	21 - 35	2	4	0	0	6
	36 - 50	6	10	0	1	17
	51 - 65	1	9	1	0	11
	> 65	0	7	5	7	19
Result: 37.7% of the originally grouped cases were classified correctly.						

a Classification of renal glomerulosclerosis: left side, female

Selection: Left side, male		Predicted group membership (in percent of sclerotic glomeruli)				
		No sclerosis	>0% - 5%	>5% - 10%	> 10%	Total
True group membership (Age in years)	21 - 35	24	6	0	0	30
	36 - 50	6	18	1	0	25
	51 - 65	2	23	10	1	36
	> 65	0	18	17	4	39
Result: 43.1% of the originally grouped cases were classified correctly.						

b Classification of renal glomerulosclerosis: left side, male

Selection:<br><br>Right side, female		Predicted group membership<br><br>(in percent of sclerotic glomeruli)				
		No sclerosis	>0% - 5%	>5% - 10%	> 10%	Total
True group membership<br><br>(Age in years)	21 - 35	3	3	0	0	6
	36 - 50	6	10	0	1	17
	51 - 65	1	7	2	1	11
	> 65	2	6	6	5	19
Result: 37.7% of the originally grouped cases were classified correctly.						

c Classification of renal glomerulosclerosis: right side, female

selection:<br><br>Right side, male		Predicted group membership<br><br>(in percent of sclerotic glomeruli)				
		No sclerosis	>0% - 5%	>5% - 10%	> 10%	Total
True group membership<br><br>(Age in years)	21 - 35	22	8	0	0	30
	36 - 50	7	17	2	0	25
	51 - 65	2	24	8	2	36
	> 65	1	18	14	6	39
Result: 40.8% of the originally grouped cases were classified correctly.						

d Classification of renal glomerulosclerosis: right side, male
